# Supplementary material for: Modeling glioblastoma heterogeneity as a dynamic network of cell states
Source: Mol Syst Biol. 2021 Sep 16;17(9):e10105. doi: 10.15252/msb.202010105 (PMC8444284; doi:10.15252/msb.202010105)
Supplement: Supplementary file 5 — Source Data for Figure 3 [file MSB-17-e10105-s001.zip › Figure3A_sourcedata/GSEA_3065/nosigdb_state3.GseaPreranked.1622718426028/LARSSON_STATE_2.html]

Details for gene set LARSSON\_STATE\_2[GSEA]

|  || Dataset | state3 |
| Phenotype | NoPhenotypeAvailable |
| Upregulated in class | na\_pos |
| GeneSet | LARSSON\_STATE\_2 |
| Enrichment Score (ES) | 0.90818846 |
| Normalized Enrichment Score (NES) | 3.4976404 |
| Nominal p-value | 0.0 |
| FDR q-value | 0.0 |
| FWER p-Value | 0.0 |
Table: GSEA Results Summary

  

Fig 1: Enrichment plot: LARSSON\_STATE\_2      
 Profile of the Running ES Score & Positions of GeneSet Members on the Rank Ordered List

  

| PROBE | GENE SYMBOL | GENE\_TITLE | RANK IN GENE LIST | RANK METRIC SCORE | RUNNING ES | CORE ENRICHMENT || 1 | BCAN |  |  | 0 | 0.883 | 0.0300 | Yes |
| 2 | SCRG1 |  |  | 3 | 0.645 | 0.0517 | Yes |
| 3 | HSP90B1 |  |  | 7 | 0.563 | 0.0704 | Yes |
| 4 | PDIA6 |  |  | 8 | 0.549 | 0.0891 | Yes |
| 5 | B2M |  |  | 11 | 0.538 | 0.1071 | Yes |
| 6 | SPCS2 |  |  | 12 | 0.532 | 0.1252 | Yes |
| 7 | MTRNR2L12 |  |  | 13 | 0.532 | 0.1433 | Yes |
| 8 | PTPRZ1 |  |  | 14 | 0.532 | 0.1614 | Yes |
| 9 | PLAT |  |  | 18 | 0.513 | 0.1785 | Yes |
| 10 | APLP2 |  |  | 20 | 0.506 | 0.1955 | Yes |
| 11 | LMAN1 |  |  | 21 | 0.496 | 0.2124 | Yes |
| 12 | PTN |  |  | 22 | 0.493 | 0.2291 | Yes |
| 13 | CD63 |  |  | 26 | 0.480 | 0.2451 | Yes |
| 14 | CANX |  |  | 27 | 0.479 | 0.2614 | Yes |
| 15 | PGRMC1 |  |  | 29 | 0.473 | 0.2773 | Yes |
| 16 | CALR |  |  | 30 | 0.473 | 0.2934 | Yes |
| 17 | LAPTM4B |  |  | 31 | 0.473 | 0.3094 | Yes |
| 18 | CD9 |  |  | 32 | 0.473 | 0.3255 | Yes |
| 19 | GPM6A |  |  | 37 | 0.460 | 0.3406 | Yes |
| 20 | GRIA2 |  |  | 38 | 0.460 | 0.3563 | Yes |
| 21 | SQLE |  |  | 39 | 0.457 | 0.3718 | Yes |
| 22 | PRDX4 |  |  | 41 | 0.455 | 0.3871 | Yes |
| 23 | HSPA5 |  |  | 43 | 0.447 | 0.4022 | Yes |
| 24 | CALU |  |  | 48 | 0.437 | 0.4166 | Yes |
| 25 | APP |  |  | 49 | 0.437 | 0.4314 | Yes |
| 26 | FGFBP3 |  |  | 51 | 0.431 | 0.4459 | Yes |
| 27 | NCAM1 |  |  | 52 | 0.430 | 0.4605 | Yes |
| 28 | PDIA3 |  |  | 55 | 0.419 | 0.4745 | Yes |
| 29 | MALAT1 |  |  | 57 | 0.412 | 0.4884 | Yes |
| 30 | PON2 |  |  | 63 | 0.389 | 0.5010 | Yes |
| 31 | ATP6AP2 |  |  | 69 | 0.380 | 0.5134 | Yes |
| 32 | TMBIM6 |  |  | 73 | 0.372 | 0.5256 | Yes |
| 33 | HMGCR |  |  | 77 | 0.369 | 0.5378 | Yes |
| 34 | SSR4 |  |  | 78 | 0.368 | 0.5503 | Yes |
| 35 | LRPAP1 |  |  | 81 | 0.361 | 0.5624 | Yes |
| 36 | FUS |  |  | 87 | 0.358 | 0.5740 | Yes |
| 37 | SOX6 |  |  | 88 | 0.358 | 0.5861 | Yes |
| 38 | HACD3 |  |  | 89 | 0.356 | 0.5982 | Yes |
| 39 | GPC1 |  |  | 90 | 0.355 | 0.6102 | Yes |
| 40 | MDM4 |  |  | 92 | 0.351 | 0.6221 | Yes |
| 41 | KCNQ1OT1 |  |  | 99 | 0.347 | 0.6332 | Yes |
| 42 | SGCE |  |  | 101 | 0.344 | 0.6447 | Yes |
| 43 | GPM6B |  |  | 105 | 0.343 | 0.6560 | Yes |
| 44 | DDOST |  |  | 106 | 0.343 | 0.6677 | Yes |
| 45 | RCN2 |  |  | 110 | 0.336 | 0.6788 | Yes |
| 46 | ACSL3 |  |  | 130 | 0.319 | 0.6874 | Yes |
| 47 | SNTG1 |  |  | 140 | 0.314 | 0.6970 | Yes |
| 48 | LAMP2 |  |  | 154 | 0.309 | 0.7060 | Yes |
| 49 | VMP1 |  |  | 156 | 0.308 | 0.7164 | Yes |
| 50 | ADGRV1 |  |  | 165 | 0.303 | 0.7257 | Yes |
| 51 | ADGRG1 |  |  | 168 | 0.301 | 0.7357 | Yes |
| 52 | PRH1 |  |  | 184 | 0.293 | 0.7439 | Yes |
| 53 | DDX17 |  |  | 191 | 0.290 | 0.7531 | Yes |
| 54 | XRCC2 |  |  | 192 | 0.290 | 0.7630 | Yes |
| 55 | NKTR |  |  | 202 | 0.282 | 0.7715 | Yes |
| 56 | LAPTM4A |  |  | 208 | 0.278 | 0.7804 | Yes |
| 57 | COL4A2 |  |  | 232 | 0.271 | 0.7869 | Yes |
| 58 | DDX39B |  |  | 236 | 0.269 | 0.7957 | Yes |
| 59 | COL4A1 |  |  | 241 | 0.267 | 0.8043 | Yes |
| 60 | TNC |  |  | 249 | 0.264 | 0.8125 | Yes |
| 61 | MARCH6 |  |  | 250 | 0.264 | 0.8215 | Yes |
| 62 | MEG3 |  |  | 258 | 0.260 | 0.8295 | Yes |
| 63 | NOVA1 |  |  | 270 | 0.253 | 0.8368 | Yes |
| 64 | ARGLU1 |  |  | 280 | 0.250 | 0.8443 | Yes |
| 65 | ZNF708 |  |  | 284 | 0.249 | 0.8524 | Yes |
| 66 | LUC7L3 |  |  | 289 | 0.247 | 0.8603 | Yes |
| 67 | SF1 |  |  | 327 | 0.235 | 0.8641 | Yes |
| 68 | ZNF431 |  |  | 345 | 0.231 | 0.8699 | Yes |
| 69 | TMEM259 |  |  | 354 | 0.229 | 0.8768 | Yes |
| 70 | ZNF75A |  |  | 374 | 0.224 | 0.8822 | Yes |
| 71 | KANSL1 |  |  | 412 | 0.216 | 0.8852 | Yes |
| 72 | MSMO1 |  |  | 413 | 0.215 | 0.8926 | Yes |
| 73 | WSB1 |  |  | 435 | 0.212 | 0.8973 | Yes |
| 74 | DDR1 |  |  | 454 | 0.208 | 0.9023 | Yes |
| 75 | LENG8 |  |  | 485 | 0.201 | 0.9057 | Yes |
| 76 | TTC14 |  |  | 522 | 0.196 | 0.9082 | Yes |
| 77 | PCSK7 |  |  | 665 | 0.176 | 0.8978 | No |
| 78 | SLC1A3 |  |  | 741 | 0.165 | 0.8948 | No |
| 79 | NEAT1 |  |  | 855 | 0.152 | 0.8869 | No |
| 80 | CREBZF |  |  | 985 | 0.139 | 0.8767 | No |
| 81 | ANKRD11 |  |  | 996 | 0.138 | 0.8803 | No |
| 82 | SPPL2B |  |  | 1235 | 0.119 | 0.8569 | No |
| 83 | MIR99AHG |  |  | 1246 | 0.118 | 0.8597 | No |
| 84 | FTX |  |  | 1541 | 0.101 | 0.8292 | No |
| 85 | MIAT |  |  | 2016 | 0.075 | 0.7771 | No |
Table: GSEA details [plain text format]

  

Fig 2: LARSSON\_STATE\_2: Random ES distribution      
 Gene set null distribution of ES for **LARSSON\_STATE\_2**
